# Long-term, Dynamic Remodelling of the Corticotroph Transcriptome and Excitability After a Period of Chronic Stress

**DOI:** 10.1210/endocr/bqae139

**Published:** 2024-10-18

**Authors:** Peter J Duncan, Nicola Romanò, Sooraj V Nair, Heather McClafferty, Paul Le Tissier, Michael J Shipston

**Affiliations:** Centre for Discovery Brain Science, Edinburgh Medical School: Biomedical Sciences, University of Edinburgh, Edinburgh EH8 9AG, UK; Centre for Discovery Brain Science, Edinburgh Medical School: Biomedical Sciences, University of Edinburgh, Edinburgh EH8 9AG, UK; Zhejiang University-University of Edinburgh Joint Institute, Zhejiang University School of Medicine, Haining 314400, PR China; Centre for Discovery Brain Science, Edinburgh Medical School: Biomedical Sciences, University of Edinburgh, Edinburgh EH8 9AG, UK; Centre for Discovery Brain Science, Edinburgh Medical School: Biomedical Sciences, University of Edinburgh, Edinburgh EH8 9AG, UK; Centre for Discovery Brain Science, Edinburgh Medical School: Biomedical Sciences, University of Edinburgh, Edinburgh EH8 9AG, UK; Zhejiang University-University of Edinburgh Joint Institute, Zhejiang University School of Medicine, Haining 314400, PR China; Centre for Discovery Brain Science, Edinburgh Medical School: Biomedical Sciences, University of Edinburgh, Edinburgh EH8 9AG, UK; Zhejiang University-University of Edinburgh Joint Institute, Zhejiang University School of Medicine, Haining 314400, PR China

**Keywords:** stress, anterior pituitary, hypothalamic pituitary adrenal axis, corticotroph

## Abstract

Chronic stress results in long-term dynamic changes at multiple levels of the hypothalamic-pituitary-adrenal (HPA) axis resulting in stress axis dysregulation with long-term impacts on human and animal health. However, the underlying mechanisms and dynamics of altered of HPA axis function, in particular at the level of pituitary corticotrophs, during a period of chronic stress and in the weeks after its cessation (defined as “recovery”) are very poorly understood. Here, we address the fundamental question of how a period of chronic stress results in altered anterior pituitary corticotroph function and whether this persists in recovery, as well as the transcriptomic changes underlying this. We demonstrate that, in mice, spontaneous and corticotrophin-releasing hormone-stimulated electrical excitability of corticotrophs, essential for ACTH secretion, is suppressed for weeks to months of recovery following a period of chronic stress. Surprisingly, there are only modest changes in the corticotroph transcriptome during the period of stress, but major alterations occur in recovery. Importantly, although transcriptional changes for a large proportion of mRNAs follow the time course suppression of corticotroph excitability, many other genes display highly dynamic transcriptional changes with distinct time courses throughout recovery. Taken together, this suggests that chronic stress results in complex dynamic transcriptional and functional changes in corticotroph physiology, which are highly dynamic for weeks following cessation of chronic stress. These insights provide a fundamental new framework to further understand underlying molecular mechanisms as well approaches to both diagnosis and treatment of stress-related dysfunction of the HPA axis.

Lifelong health and wellbeing are critically dependent on the ability of an organism to respond appropriately to stress in response to a changing environment (1-5). Stress increases the output of glucocorticoid hormones, regulated by the neuroendocrine hypothalamic-pituitary-adrenal (HPA) axis. Glucocorticoids have a major impact on a range of physiological responses but also limit HPA axis activity by negative feedback at multiple levels, including the brain and anterior pituitary corticotroph (6-9).

Despite the homeostatic impact of negative feedback, sustained physiological or pathophysiological activation of the HPA axis, such as in chronic stress or trauma, can lead to dysregulation of the HPA axis (10-12). Importantly, dysregulation of the HPA axis can occur both during the exposure to chronic stress as well as for weeks to months after the cessation of the chronic stress (defined here as “recovery”). HPA axis dysregulation can typically be characterized by distinct phases as a result of dissociation between the output of ACTH from pituitary corticotrophs and glucocorticoids from the adrenal fasciculata cells. For example, in humans, ACTH secretion remains blunted for several weeks after several different types of chronic stressor even though cortisol output returns to prestress levels and it takes several months for both ACTH and cortisol responses to normalize (2-4, 11, 12) Although several mechanisms have been proposed to contribute (10-12), the role of the anterior pituitary corticotroph in these dynamic changes is largely unexplored, even though the use of corticotrophin-releasing hormone (CRH)-stimulation tests in humans reveals the critical role of CRH-driven mechanisms at the level of the anterior pituitary.

Corticotrophs are electrically excitable and their functional response to CRH is determined by a transition in their pattern of electrical excitability from a single spiking mode to pseudo plateau bursting. In male rats, a 14-day exposure to chronic variable stress immediately results in sensitization of corticotrophs (13), as revealed by enhanced basal and CRH-evoked ACTH secretion in isolated perifused pituitaries in vitro from CVS rats compared to control. Using a 14-day repeated daily restraint stress paradigm in male mice, we previously showed sensitization of the corticotroph because of elevated basal and CRH-induced electrical bursting behavior in isolated corticotrophs in vitro (14). However, whether these changes in excitability recover or are long-lasting following removal of the stress is unknown. Moreover, changes in corticotroph excitability and function in response to chronic stress are presumed to be driven by changes in corticotroph gene expression (9, 15, 16). However, the dynamics of gene expression at the level of the corticotroph, pathways controlled that may regulate corticotroph physiology, or whether chronic stress induces long-term changes in gene expression that are sustained in recovery are not known. Thus, a key question in pituitary biology is whether chronic stress results in long-term changes in the corticotroph transcriptome and/or excitability that remain even after the chronic stress is removed or whether corticotrophs rapidly adapt following the cessation of chronic stress.

In this study, we have used a well-established 14-day chronic repeated daily restraint stress model in mice (14, 17) to examine how the transcriptome and electrical excitability of corticotrophs is modified after exposure to chronic stress and following removal of the stressor. We reveal highly dynamic changes in both the corticotroph transcriptome and excitability of corticotrophs. We observe a major suppression of gene expression and excitability 4 weeks following the cessation of the period of chronic stress that partially recovers by 3 months. However, we also see major changes in gene expression at 3 months, distinct from those genes whose expression is modified at 4 weeks of recovery.

These data reveal fundamental insights into the dynamics of stress biology at the level of the corticotroph and reveal new mechanistic insight into the suppression of corticotroph function after a period of chronic stress important for the diagnosis and treatment of chronic stress related disorders.

## Materials and Methods

### Reagents

General biochemical reagents used throughout this study were obtained from Sigma-Aldrich (St. Louis, MO, USA) and were of analytical grade quality unless stated otherwise.

### Animals

Mice expressing green fluorescent protein (GFP) under the proopiomelanocortin (POMC) promoter, were used as previously described (18) on a C57/BL6 background. Mice were caged in groups of 2 to 4 under standard laboratory conditions (lights on at 7:00 Am, lights off at 7:00 Pm, 21 °C, with tap water and chow available ad libitum) at the University of Edinburgh. Male mice aged 2 to 7 months were used for pituitary cell culture from tissue collected between 9:00 Am and 10:00 Am following cervical dislocation. All breeding and tissue collection were performed in accordance with UK Home Office requirements (PPL PP2870833) and University of Edinburgh Ethical Review Committee approval (PL26-21).

### Chronic Stress Protocol

We used a well-established 14-day repeated daily restraint stress paradigm as our chronic stress protocol that in male C57/Bl6 mice induces anxiety and depression-like behavior along with reduction in body weight and elevated basal blood corticosterone levels (14, 17). This homotypic chronic stress model results in a modest habituation of the HPA axis (as determined by blood corticosterone in response to the daily restraint) but mice still mount a robust acute stress response on day 14 that is >70% of the response on the first day of the protocol. In addition, the model results in sensitization of the corticotroph at the end of the 14-day chronic stress period (14) as also observed using chronic variable stress over 14 days in rats (13). This 14-day repeated daily restraint stress model was chosen over other commonly used models in rodents, such as chronic variable stress using multiple stressors randomly per day or chronic social defeat stress paradigms because it is a simple, highly reproducible, and tractable model of chronic stress that results in robust changes at the molecular, cellular, systems, and behavioral level in mice without subjecting mice to painful stressors (17).

Mice were subjected to 30 minutes of restraint stress each day for 14 days. For the restraint groups, mice were placed individually in clear plastic restraint tubes (CH Technologies [USA] Inc., Westwood, NJ, USA) of internal diameter 31 mm with a variable pusher, adjusted based on animal size. Tissue was either collected on day 15 or mice were allowed to recover for between 1 and 16 weeks.

Mice were randomly assigned to 1 of 6 experimental groups. There was a group of control, nonstressed (CTRL) mice. There was a group of mice subjected to chronic stress (CS) for 14 days with tissue collected on day 15. There was a set of 4 recovery groups in which mice were subjected to 14 days of restraint stress and allowed to recover for 1 week (CS-1R), 4 weeks (CS-4R), 12 weeks (CS-12R), or 16 weeks (CS-16R). In addition, the contribution of age was investigated by comparing CTRL groups with data obtained from unstressed mice that were age-matched to CS-12R groups (designated “aged-CTRL”). The mean age ± SD (in weeks) at time of tissue collection for each group (combined electrophysiology and RNA-sequencing [RNA-seq] experiments) was: CTRL—13.8 ± 0.86, CS—14.3 ± 0.75, CS-1R—16.4 ± 0.78, CS-4R—16.1 ± 1.03, CS-12R—26.0 ± 0.53, CS-16R—30.4 ± 0 (single prep), and aged-CTRL—25.6 ± 2.06.

### Cell Culture

Anterior pituitary cells were acutely isolated by trypsin digestion as previously described (18). Each cell preparation used anterior pituitary glands collected from 3 animals. For electrophysiological experiments, cells were cultured on 12-mm coverslips (Warner Instruments, Holliston, MA, USA) in serum-free medium (Dulbecco's modified Eagle's medium containing 25 mM HEPES, 5 μg/mL insulin, 50 μg/mL transferrin, 30 nM sodium selenite, 0.3% BSA [w/v], 4.2 μg/mL fibronectin, and antibiotic/antimycotic [100× dilution of Sigma stock]) and incubated at 37 °C in 5% CO2. Serum-free medium (lacking antibiotic/antimycotic) was changed every 2 days and electrophysiological recordings were obtained from cells 24 to 96 hours after isolation.

### Electrophysiology

Electrophysiological recordings were obtained from GFP-identified corticotroph cells using the perforated patch mode of the whole-cell patch clamp technique. Amphotericin B was used at a concentration of 150 μg/mL in pipette solution, which resulted in access resistances typically less than 40 MΩ within 10 to 20 minutes and allowed stable recordings in excess of 40 minutes. The standard bath solution (extracellular) contained the following (in mM): 140 NaCl, 5 KCl, 2 CaCl_2_, 0.1 MgCl_2_, 10 HEPES, and 10 glucose. The pH and osmolality were adjusted to 7.4 with NaOH and 300 mOsmol/L, respectively. The standard pipette solution (intracellular) contained the following (in mM): 10 NaCl, 30 KCl, 60 K_2_SO_4_, 1 MgCl_2_, 10 HEPES, 10 glucose, and 50 sucrose. The pH was and osmolality were adjusted to 7.3 with KOH and 290 mOsmol/L, respectively. Recordings were performed at room temperature (18-22 °C) to facilitate stable recordings of more than 30 minutes required for these assays and obtained using Clampex 10.7 (Molecular Devices, San Jose, CA, USA) with a sampling rate of 10 kHz and filtered at 2 kHz. Patch pipettes were fabricated from borosilicate glass (King Precision Glass, Inc., Claremont, CA, USA) using a model P-97 micropipette puller (Sutter Instrument Co., Novato, CA, USA). Pipette tips were heat polished and had resistances typically between 2 and 3 MΩ. Compensated series resistance was typically less than 20 MΩ and capacitance of corticotrophs ranged from 2 to 14 pF. A gravity-driven perfusion system was used to apply drugs to the cells with a flow rate of 1 to 2 mL/min to minimize flow-induced artifacts.

### Dynamic Clamp

Dynamic clamp experiments were performed using a separate digital acquisition card and computer running the software QuB (19). In the current clamp mode of the patch amplifier (Axopatch 200B; Molecular Devices), membrane potential *V* was used to compute the current going through the large conductance calcium- and voltage- activated potassium (BK) channels, $I_{BK}=g_{BK}f(V_{K}-V)$ with *f* obtained by integrating


$$\tau_{BK}\frac{df}{dt}=f_{\infty }(V)-f$$

in real time using the forward Euler method (19), with an average time step of 21 μs (maximum ≤100 μs), and the steady-state BK channel activation given by


$$f_{\infty }(V)={\left[1+exp\left(\frac{\left(v_{f}-V\right)}{s_{f}}\right)\right]}^{-1}$$

The calculated BK current was injected back into the cell through the same digital acquisition card. Typical parameter values were as follows: $g_{BK}$ = 0.5 to 2 nS; $v_{f}$ = −10 mV; $s_{f}$ = 2 mV; $\tau_{BK}$ = 2 ms. However, because of the intrinsic variability of corticotroph activity, parameters were modified slightly from cell to cell.

### Electrophysiological Analysis

Current clamp recordings were analyzed as previously described (14, 18, 20, 21) using Clampfit v.10.7 (Molecular Devices). Corticotroph excitability was measured for a minimum of 60 seconds under basal conditions and during CRH-evoked activity, which was measured immediately after 3 minutes of CRH stimulation (0.2 nM). The membrane potential was calculated by averaging 3 time points at the beginning, middle, and end of the measurement period. Properties of spikes and bursts (collectively, “events”) were measured using the Event Detection function of Clampfit software and manually verified. An event was defined from the point the membrane potential reached the threshold (Δ20 mV from baseline) until it fell below a rearm level (Δ5 mV). This allowed the calculation of mean event frequency and duration. In addition, bursting behavior was quantified through the calculation of a burst factor. This method classifies any event <100 ms in duration as a spike, and events >100 ms duration, which also have at least 2 spikelets during the event, as a burst. A burst factor was calculated as the proportion of the number of total events that are bursts (14, 18, 20, 22).

Data in the text are presented as the means ± SD, where n represents the number of cells from across multiple independent preparations of cells, with each preparation normally generated from three animals. The data in figures are presented as box plots divided into quartiles and overlaid with data points from individual cells. Statistical analyses of electrophysiological parameters were performed using R 4.0.5. Quantitative data were analyzed using linear regression when data points were independent, or linear mixed-effect models when multiple measurements were taken from the same cell, in which case the cell was used as a random effect. Event frequency and event duration were log-transformed to meet the assumption of normality for the model residuals (for the frequency) and to correct for heteroscedasticity (for the duration); all other model assumptions were met. Post hoc comparisons were performed using the Tukey test when the main effects or interactions were found to be significant. Significant differences between groups were defined (*P < .05 and **P < .01).

### RNA-seq Sample Preparation and Sequencing

Pituitaries were isolated and enzymatically dissociated as described for the electrophysiology experiments, using three pituitaries per independent sample. After dissociation, the cells were resuspended in 500 μL of PBS supplemented with 25 mM HEPES and 5 mM EDTA (fluorescence-activated cell sorting [FACS] buffer) and passed through a 35-μm cell strainer that was further washed with 200 μL of the FACS buffer. Draq7 was added as a vitality marker, and cells were sorted using an SH800 cell sorter (Sony). Gates were established using wild-type pituitaries to avoid capturing enhanced green fluorescent protein (eGFP)-negative (−ve) cells and to select single cells. Sorted single cells were resuspended into low-bind Eppendorf tubes into 300 μL of Trizol, frozen on dry ice, and stored at −80 °C until sending for sequencing.

RNA isolation, library preparation, and sequencing reactions were conducted at Azenta Life Sciences (South Plainfield, NJ). Total RNA was extracted using the QIAGEN RNeasy Plus Mini Kit following the manufacturer's instructions (QIAGEN, Hilden, Germany). The extracted RNA samples were quantified using the Qubit Fluorometer (Life Technologies, Carlsbad, CA, USA), and RNA integrity was checked using the Agilent TapeStation (Agilent Technologies, Palo Alto, CA, USA). An ultra-low-input RNA sequencing library was prepared by using the SMART-Seq v4 Ultra Low Input Kit for Sequencing for full-length cDNA synthesis and amplification (Clontech, Mountain View, CA, USA), and the Illumina Nextera XT (Illumina, San Diego, CA, USA) library was used for sequencing library preparation. Then, cDNA was fragmented, and an adaptor was added using transposase, followed by limited-cycle PCR to enrich and add an index to the cDNA fragments. The sequencing library was validated on the Agilent TapeStation (Agilent Technologies) and quantified by using a Qubit Fluorometer (Thermo Fisher Scientific, Waltham, MA, USA), as well as by quantitative PCR (KAPA Biosystems, Wilmington, MA, USA). The sequencing libraries were multiplexed and clustered onto a flowcell. After clustering, the flowcell was loaded onto the Illumina HiSeq instrument according to the manufacturer's instructions. The samples were sequenced using a 2 × 150-bp paired-end configuration. Image analysis and base calling were conducted using HiSeq Control Software. Raw sequence data (.bcl files) generated from Illumina HiSeq were converted into fastq files and demultiplexed using Illumina bcl2fastq 2.17 software. One mismatch was allowed for index sequence identification.

### RNA-seq Bioinformatics Analysis

Quality of the fastq files was assessed using FastQC v0.11.9 (https://www.bioinformatics.babraham.ac.uk/projects/fastqc/); all samples were satisfactory. Reads were aligned to the mouse genome (GRCm39 release 107) using STAR v2.7.10a (https://github.com/alexdobin/STAR). The aligned bam files were processed using SummarizeOverlaps from the GenomicAlignments Bioconductor package (23) in R 4.2.3.

RNA-seq data were obtained from 2 separate rounds of sequencing. The first round contained CTRL, CS, and CS-4R group (all n = 3 independent samples; each sample includes pituitaries from 3 mice). Data from this CTRL group have been previously published alongside control female data (21). The second round contained additional CTRL and CS-12R cells (both n = 3), together with additional age-matched controls for the CS-12R group. The count matrices of the datasets from the 2 experiments were merged and the count were adjusted using ComBat-Seq (24) to remove batch effects. The combined counts were then analyzed using DESeq2 (25), keeping only genes with a count of 10 or higher in at least 2 of 3 independent samples. Values of log2-fold changes were shrunk using an approximate posterior estimation for general linear model (GLM) from the apeglm package (26) before performing the Wald test to determine differentially expressed genes. The reported *P* values are corrected for multiple comparisons using the Benjamini-Hochberg false discovery rate correction. To group genes depending on their temporal profile, we considered all genes differentially expressed between any 2 groups. We performed unsupervised clustering of the gene expression of these genes using Ward's method, then cutting the obtained dendrogram to obtain the 4 most prominent groups. Gene ontology (GO) pathway analysis of the genes in these 4 groups was performed using ShinyGo (27) with tables of enriched GO terms in Supplementary Tables S1 to S3 (28). The code used for these analyses is available at https://github.com/nicolaromano/Duncan2024-ChronicStress/; raw data are available on EBI (E-MTAB-13648). An interactive dashboard to explore the sequencing results is available at https://apps.nicolaromano.net/CorticotrophsRNAseq/ChronicStress.

## Results

### Corticotroph Excitability Is Suppressed for Several Weeks After the Cessation of a Period of Chronic Stress

**Figure 1.**
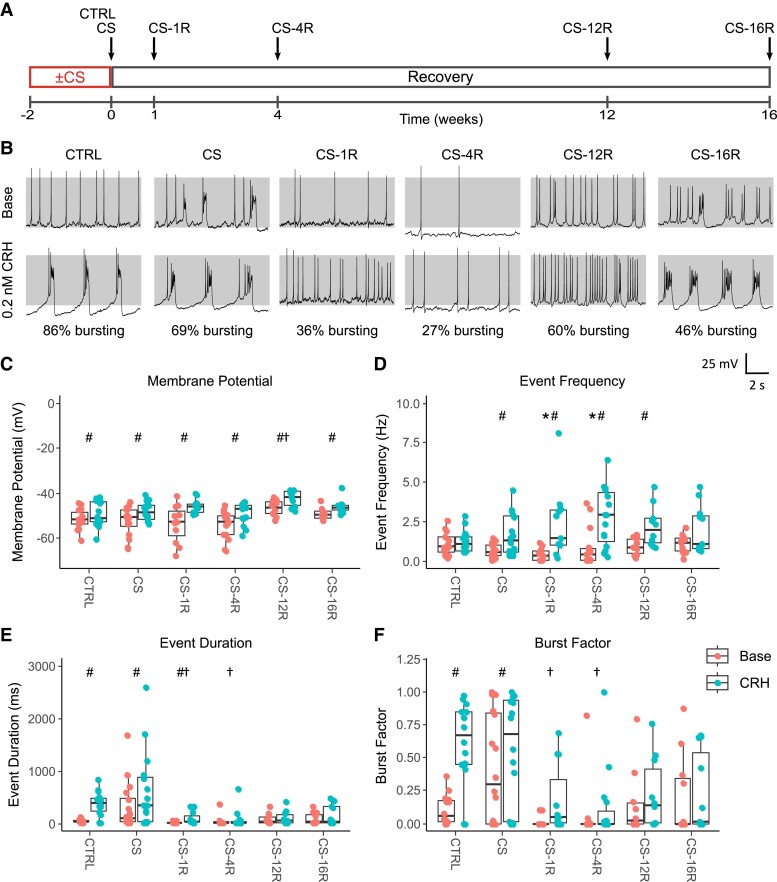
Electrical excitability is modulated in chronic stress and recovery. (A) Timeline of chronic stress paradigm, in which mice were subjected to 30 minutes of restraint stress per day for 2 weeks, with or without a recovery period. Groups for electrophysiological experiments included unstressed controls (CTRL, n = 14 cells), mice sacrificed immediately following chronic stress (CS, n = 16 cells), and mice subjected to chronic stress plus a 1- (CS-1R, n = 11 cells), 4- (CS-4R, n = 15 cells), 12- (CS-12R, n = 10 cells), or 16-week (CS-16R, n = 11 cells) recovery period. (B) Representative traces of basal and CRH-evoked activity (0.2 nM for 3 minutes) from each group, with percentage of cell displaying CRH-induced bursting below. Gray shading indicates membrane potential between −50 and +10 mV. (C) A CRH-induced membrane depolarization was observed in all groups, suggesting changes in frequency and bursting in stress and recovery occur independently of membrane potential. (D) Basal event frequency was significantly reduced in CS-1R and CS-4R cells compared to controls, but CRH was still able to cause a significant increase in single-spike frequency in these cells. CRH-induced bursting behavior was also altered in stress and recovery. (E) CRH-evoked event duration and (F) burst factor was significantly reduced in CS-1R and CS-4R cells compared to controls. ^#^Group significantly different base vs CRH; *Base significantly different vs control; ^†^CRH significantly different vs control (*P* < .05, linear mixed-effects model [lme], with Tukey post hoc test). n represents number of cells, from pituitary preparations each using 3 animals.

Our previous work has revealed that a 2-week period of repeated daily restraint stress (CS), using a well-defined repeated restraint-stress model in mice, results in an increase in the proportion of murine corticotrophs that display both basal and CRH-evoked hyperexcitability (14). To address whether this hyperexcitability rapidly reverses once the period of chronic stress is removed, or alternatively if chronic stress results in long-time dynamic changes in corticotroph excitability, we analyzed basal and CRH-induced electrical activity by perforated patch-clamp electrophysiology in isolated corticotrophs from male Pomc-GFP mice. Male Pomc-GFP mice were exposed to 2 weeks of 30 minutes restraint stress daily (CS) or corresponding age-matched controls (CTRL) and mice exposed to CS and subsequently returned to the home cage for 1 week (CS-1R), 4 weeks (CS-4R), 12 weeks (CS-12R), or 16 weeks (CS-16R) (Fig. 1A).

**Figure 2.**
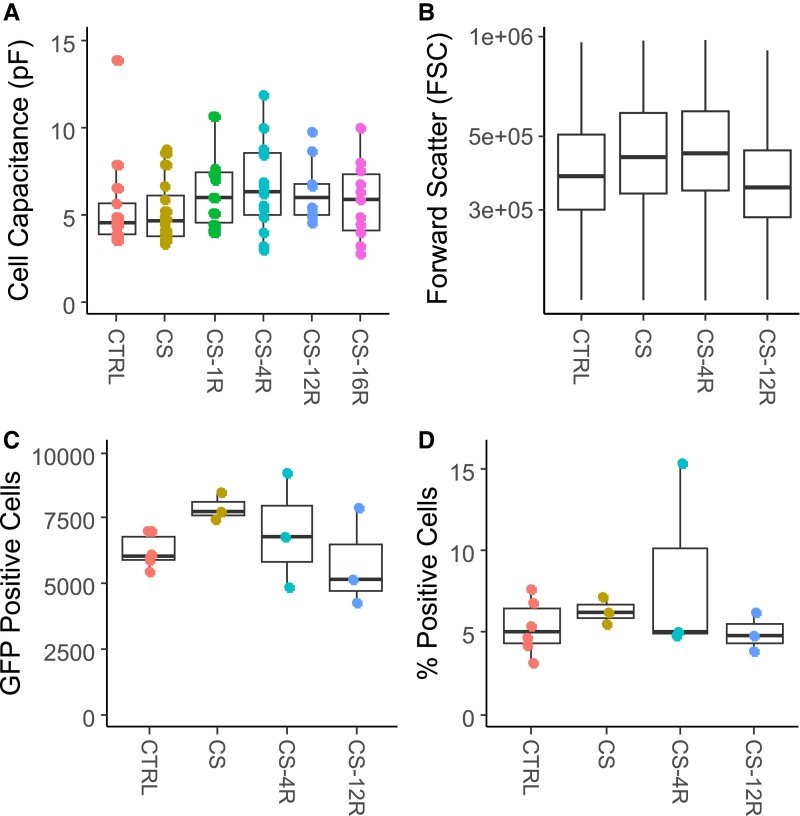
Corticotroph size and number is not altered by chronic stress. (A) Cell capacitance from electrophysiological recordings, which is proportional to cell surface are, showed no significant difference between CTRL (n = 14 cells), CS (n = 16 cells), CS-1R (n = 11 cells), CS-4R (n = 15 cells), CS-12R (n = 10 cells), and CS-16R cells (n = 11 cells). For RNA-seq experiments, corticotrophs were isolated by FAC sorting from cells pooled from anterior pituitaries of 3 Pomc-GFP mice. (B) Forward scatter (FSC), which is proportional to cell size, is not significantly different between CTRL (n = 6 preps), CS (n = 3 preps), CS-4R (n = 3 preps), and CS-12R cells (n = 3 preps). In addition, there was no significant change in the number or proportion of corticotrophs in the anterior pituitary. (C) The total number of GFP positive cells and (D) as a percentage of total live cells was not changed in stress and recovery. Data were analyzed using linear regression (lm).

As previously reported (14, 18), control male mouse corticotrophs (n = 14 cells) display spontaneous low-frequency action potentials (1.08 ± 0.72 Hz) with the majority (>95%) of cells displaying a transition to bursting upon stimulation with physiological levels (0.2 nM) of CRH (Fig. 1B). The resultant increase in event duration (t_71_ = −7.999; *P* < 0.0001) from 44 ± 30 ms to 379 ± 234 ms (Fig. 1E) and burst factor (t_71_ = −7.988; *P* < .0001) from 0.11 ± 0.11 to 0.61 ± 0.32 (Fig. 1F) is indicative of bursting in these cells. As we have shown previously (14), after 2 weeks of chronic stress (n = 16 cells), corticotrophs display spontaneous hyperexcitability (CS, Fig. 1B) with increased basal burst factor (0.39 ± 0.41) that is not a result of changes in unstimulated resting membrane potential (Fig. 1C) or cell capacitance (Fig. 2A). However, in CS cells, CRH can still drive bursting, a mechanism that is dependent upon function large-conductance calcium- and voltage-activated BK channels (14, 18).

However, 1 week after the period of chronic stress was removed (CS-1R; n = 11 cells), corticotrophs showed a reduced spontaneous activity manifest as both a significant (t_71_ = 3.748; *P* = .042) reduction in spontaneous spiking frequency (0.40 ± 0.41 Hz) and a decrease in basal burst factor (0.02 ± 0.04) compared to both CS and control corticotrophs (Fig. 1B-1F). Remarkably, this major suppression of spontaneous excitability was maintained 4 weeks after cessation of chronic stress (CS-4R; n = 15 cells) demonstrating long-term changes to corticotroph excitability. Importantly, at both CS-1R and CS-4R, CRH-evoked bursting was also very dramatically reduced. At CS-4R, CRH only induced any bursting in less than 30% of cells and both CRH-induced event duration (80 ± 165 ms) and burst factor (0.12 ± 0.27) were significantly attenuated (t_71_ = 4.273; *P* = .0008 and t_71_ = 4.478; *P* = .0004, respectively) compared to control cells (Fig. 1B-1F). The reduced spontaneous and CRH-evoked bursting was not per se a result of significant changes in resting membrane potential or ability of CRH to induce the typical small (few mV) membrane depolarization observed in control cells (Fig. 1C). This suggests the reduced excitability observed at both CS-1R and CS-4R is not a result of changes in functional expression of conductances controlling resting membrane potential. Indeed, at CS-1R and CS-4R, although CRH bursting was largely abolished, CRH was able to significantly increase event frequency (t_71_ = −5.980; *P* < .0001 and t_71_ = −7.248; *P* < .0001, respectively; Fig. 1D) driven by an increase in the frequency of single-spike action potentials with event durations typically < 100 ms. Previous functional and modelling studies have revealed that bursting in pituitary cells is also correlated with cell size with larger cells more likely to display bursting (29). However, no significant changes in cell membrane capacitance were observed (Fig. 2A) from cells recorded at CS-1R or CS-4R compared to other groups. Moreover, in agreement with the capacitance measurements from electrophysiologically characterized corticotrophs, there were no significant differences in cell size determined from FACS in any group (Fig. 2B). This demonstrates that changes in cell size are not a major contributing factor in suppression of spontaneous or CRH-evoked bursting behavior. Importantly, we also saw a significant reduction in the number of corticotrophs that displayed any CRH-induced bursting at both CS-1R and CS-4R, suggesting that the intrinsic heterogeneity of corticotroph behavior across the population is also dynamically modified for weeks after a period of chronic stress.

To address whether this suppression of spontaneous and CRH-evoked excitability 1 to 4 weeks following removal of the stress was reversible, we analyzed spontaneous and CRH-evoked electrical activity 12 (CS-12R; n = 10 cells) and 16 (CS-16R; n = 11 cells) weeks after the period of chronic stress was removed. Spontaneous excitability had recovered to near control levels at both 12 and 16 weeks following cessation of the stress (Fig. 1B-1F). In contrast, recovery of CRH-induced bursting was only partially restored over this time domain with ∼50% of cells showing robust CRH-induced bursting. Again, at both CS-12R and CS-16R CRH was able to promote an increase in event frequency (2.19 ± 0.41 Hz and 2.02 ± 1.52 Hz) from an increase in single spike frequency.

Taken together, these data suggest that the inability to transition to bursting at CS-1R and CS-4R is not per se an inability of CRH to regulate key conductances involved in corticotroph excitability. Rather, it reveals mechanisms that control bursting are not fully engaged. Importantly, even after a short 2-week period of chronic stress CRH-induced bursting behavior is abrogated for several months after the cessation of chronic stress.

Because of the extended recovery periods in our study, the CS-12R groups were on average 12 weeks older than CTRL groups at the time of tissue collection. To investigate whether age was contributing to changes in electrical excitability, cells were isolated from unstressed mice that were aged-matched to CS-12R groups (Supplementary Fig. S1 (28)). There was no significant difference (t_20_ = −0.473; *P* = .6414) in cell capacitance of aged-CTRL cells CTRL. Additionally, there was no significant effect of age on either basal or CRH-evoked membrane potential (t_20_ = 0.990; *P* = .3338), event frequency (t_20_ = −0.089; *P* = .9303), event duration (t_20_ = 1.045; *P* = .3083), or burst factor (t_20_ = 0.229; *P* = .8216). This suggests that changes in excitability during stress and recovery occur independently of age. Full data can be seen in supplementary information (28) https://doi.org/10.7488/ds/7791.

Bursting in corticotrophs and other anterior pituitary cells is critically dependent upon the activity of large conductance calcium- and voltage-activated BK channels. Indeed, genetic deletion or pharmacological inhibition of BK channels prevents CRH-induced bursting in murine corticotrophs and bursting is dependent on only a small number of active BK channels (14, 18). Furthermore, short-term (minutes to hours) exposure of murine corticotrophs to glucocorticoid hormones results in a loss of CRH-induced bursting that can be almost completely restored by introduction of BK channel conductances using dynamic clamp (20) and the hyperexcitability of corticotrophs after 2 weeks of chronic stress is dependent upon functional BK channels (14).

**Figure 3.**
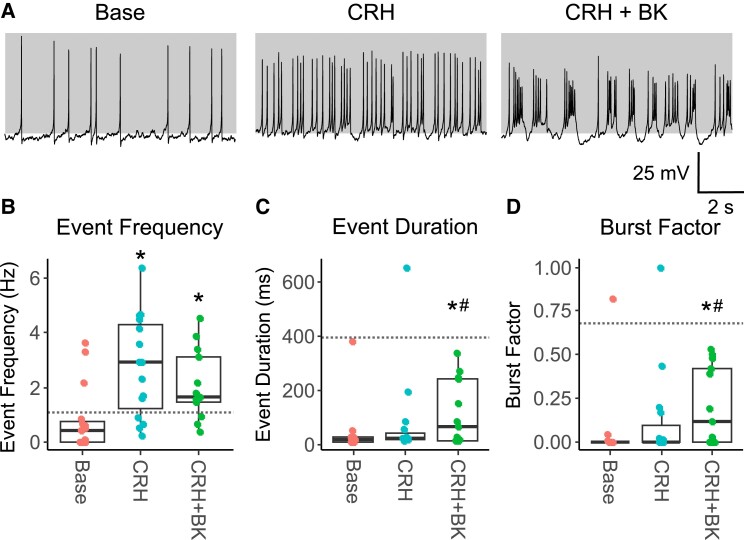
Suppression of CRH-induced bursting in CS-4R cells can be partially rescued with BK currents. (A) Representative traces from CS-4R cells under basal conditions (n = 15 cells), following stimulation with 0.2 nM CRH for 3 minutes (n = 15 cells) plus addition of BK current using dynamic clamp (n = 13 cells). Gray shading indicates membrane potential between −50 and +10 mV. (B) CRH was able in significantly increase event frequency in CS-4R cells. Although CRH-induced bursting was suppressed in CRH-4R cells, addition of BK current with dynamic clamp was able to partially rescue bursting behavior. Although (C) event duration and (D) burst factor were not significantly increased by CRH alone, addition of the BK conductance significantly increased both event duration and burst factor. However, the transition from spiking to bursting remained attenuated compared to control cells. The median CRH-evoked event frequency, event duration, and burst factor of control cells (from Fig. 1) are indicated on their respective graphs with a horizontal dotted line. The parameters were gBK = 1 nS; vf = −10 mV; sf = 2 mV; and τBK = 2 ms, but slight variations were made in some cells depending on their intrinsic properties. *Significantly different vs base; ^#^CRH + BK significantly different vs CRH (*P* < .05, linear mixed-effects model [lme], with Tukey post hoc test). n represents number of cells, from pituitary preparations each using 3 animals.

We thus reasoned that the reduced spontaneous and CRH-induced bursting in CS-1R and CS-4R corticotrophs may result from a functional uncoupling between CRH signalling and BK channels. To test this, we recorded from CS-4R corticotrophs exposed to 0.2 nM CRH and used dynamic clamp to reintroduce BK conductances (Fig. 3) that restores CRH-bursting in corticotrophs from mice in which BK channels are genetically deleted or in control corticotrophs acutely treated with glucocorticoids. Although CRH induced a significant (t_26_ = −5.720; *P* < .0001) increase in event frequency from 0.83 ± 1.21 Hz to 2.78 ± 1.85 Hz (Fig. 3B), there was no transition to bursting. Introduction of BK conductance by dynamic clamp resulted in a modest but significant (t_26_ = −3.061; *P* = .0136) increase of either event duration to 80 ± 165 ms (Fig. 3A and 3C), as well as a significant (t_26_ = −2.801; *P* = .0249) increase in burst factor to 0.12 ± 0.27 (Fig. 3A and 3D) in a proportion of CRH-treated CS-4R corticotrophs (7/13 cells). Dynamic clamp could not completely restore CRH-induced bursting and median event duration and burst factor was suppressed compared to CRH-stimulated control cells (392 ms and 0.68, respectively). Indeed, CRH-induced event frequency resulting from an increase in single spike activity was not significantly attenuated by introduction of BK conductances with dynamic clamp. This suggests that although functional uncoupling between CRH signaling and BK channels likely contributes to the reduction in bursting, other mechanisms to limit CRH-induced bursting are also involved. Moreover, the suppression of basal spontaneous action potential generation is likely to result from additional mechanism(s).

### Long-term, Dynamic Changes in Corticotroph Transcriptome Are Manifest Only Weeks Following the Cessation of a Period of Chronic Stress

**Figure 4.**
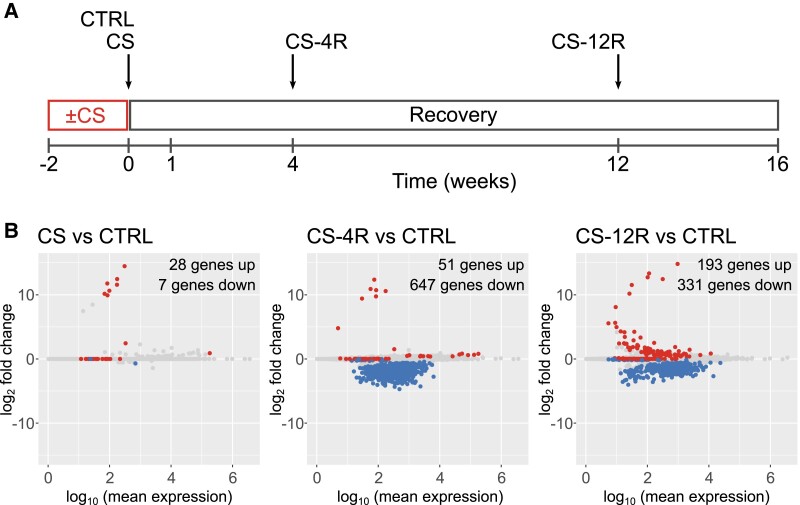
Corticotroph gene transcription is modified in chronic stress and recovery. (A) Timeline of chronic stress paradigm, where mice were subjected to 30 minutes of restraint stress per day for 2 weeks, with or without a recovery period. Groups for RNA-seq experiments included unstressed controls (CTRL, n = 6 independent preps), mice sacrificed immediately following chronic stress (CS, n = 3 preps), and mice subjected to chronic stress plus a 4- (CS-4R, n = 3 preps) or 12-week (CS-12R, n = 3 preps) recovery period. (B) MA plots reveal differentially expressed genes (*P* < .05) in CS, CS-4R, and CS-12R cells compared to CTRL. Genes above the zero line (positive values, red dots) are upregulated, whereas genes below zero line (negative values, blue dots) are downregulated in respective groups compared to controls. n represents number of independent preparations of pituitary cells each using pituitaries from 3 animals.

In an attempt to better understand the molecular mechanisms that are likely to drive these long-term dynamic changes in corticotroph electrical excitability, we determined the impact of chronic stress on corticotroph gene transcription. We performed RNA-seq on FACS corticotrophs from male Pomc-GFP mice exposed to 2 weeks of daily 30 minutes of restraint stress (CS) or corresponding age-matched controls (CTRL) and mice exposed to CS and subsequently returned to the home cage to recover for 4 weeks (CS-4R) or 12 weeks (CS-12R) (Fig. 4A). These time points represent the periods of the most dramatic changes in corticotroph excitability we observed in the electrophysiological analysis (Fig. 1). No significant differences in absolute corticotroph number or percentage of total cells (Fig. 2C and 2D) were observed between groups. Together, with the capacitance and FACS data that revealed no change in cell size (Fig. 2A and 2B); this supports that major changes in corticotroph cell mass are not responsible for the dramatic changes in excitability during or following a period of chronic stress.

Compared to controls, CS corticotrophs displayed a very modest change in gene expression (Fig. 3B, left) with only 35 significantly (adjusted *P* value < .05) differentially expressed genes (DEGs) compared to control, that is consistent with recent single-cell RNA-seq data in male mice subjected to 3 weeks of social defeat stress in which 32 DEGs were observed in the single-cell corticotroph population (16). However, in parallel with the major suppression of corticotroph excitability, mice exposed to CS and allowed to recover for 4 weeks (CS-4R) displayed extensive transcriptional changes, more than an order of magnitude larger than immediately following CS, compared to control or CS groups with 698 DEGs (Fig. 4B, middle). Significant differences in the transcriptome compared to control, was still observed at 12 weeks following the cessation of the chronic stress (524 DEGs; Fig. 4B, right). This reveals that transcriptional changes are significantly more pronounced in the period after an exposure to chronic stress with maximal differences observed after 4 weeks of cessation of the chronic stress.

**Figure 5.**
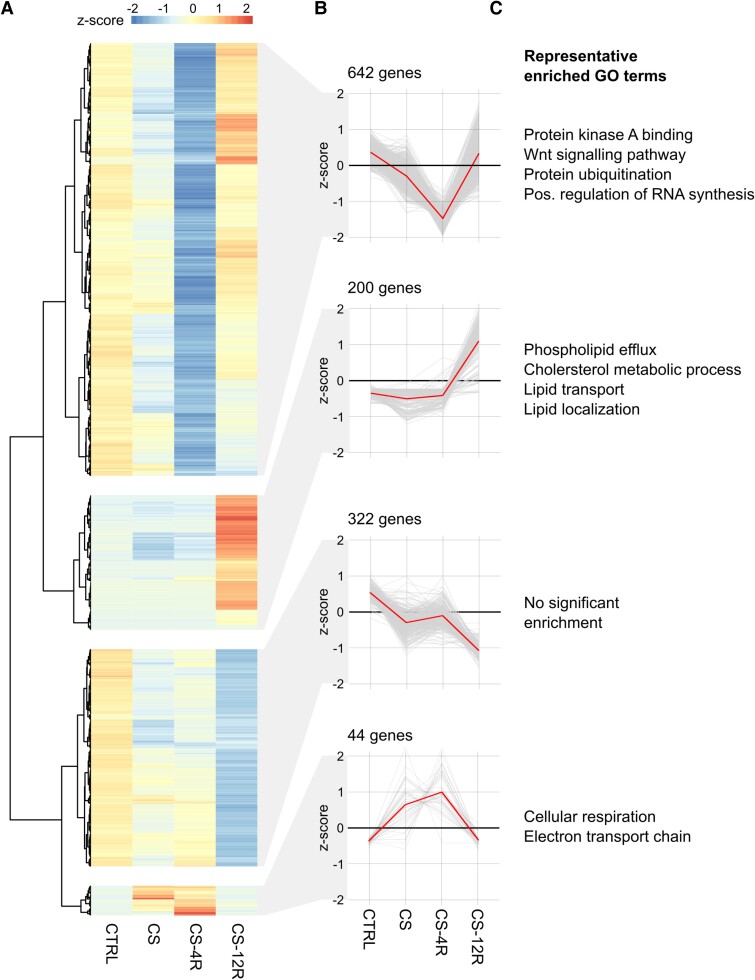
Differential gene expression profiles in stress and recovery. Corticotrophs from control (CTRL, n = 6 preps), chronic stress (CS, n = 3 preps), and stress plus 4- (CS-4R, n = 3 preps) or 12-week (CS-12R, n = 3 preps) recovery were FACS-sorted and the transcriptome analyzed by bulk RNA-seq. (A) Heatmap showing differentially expressed genes with relative expression across each row (*z*-score) is shown as per the color code. (B) Cluster analysis of DEGs profiles revealed changes in the expression profile of DEGs under each condition. Four major clusters were observed that differed in the number of genes, direction, and dynamics of mRNA changes in response to CS and with recovery. (C) GO pathway analysis revealed significantly enriched GO terms for 3 of the 4 clusters. n represents number of independent preparations of pituitary cells each using pituitaries from 3 animals.

Cluster analysis of the DEGs profiles across all conditions (Fig. 5A and 5B) revealed highly dynamic and distinct changes in the expression profile of DEGs under each condition. Four major clusters were observed that differed in both the number of genes, direction, and dynamics of mRNA changes in response to CS and following removal of the stress. The largest cluster includes 642 genes, in which mRNA expression is significantly reduced at CS-4R but largely recover expression to control levels by CS-12R. GO pathway analysis (for full GO enriched list, see Supplementary Tables S1-S3 (28)) revealed that these downregulated genes were enriched for genes involved in signal transduction pathways in particular the cAMP/protein kinase A (PKA) pathway that is critical for CRH signaling in corticotrophs (Fig. 5C). For example, genes encoding subunits of heterotrimeric G proteins (*Gnai*1, *Gnb1, Gng*4), adenylate cyclases (*Adcy*3, *Adcy*9), phosphodiesterases (*Pde3*b), catalytic and regulatory subunits of PKA (*Prkar2a*, *Prkaca*), type 2A protein Ser/Thr phosphatase family members (*Ppp2ca, Ppp6r1*), as well as downstream cAMP/PKA-dependent transcriptions factors (*Creb3l1*). In contrast, no significant changes in expression of the CRH G-protein coupled receptor expressed in corticotrophs (*Crhr1*) was observed, suggesting that CRH-dependent signaling downstream of the CRHR1 receptor may be modified. These data support the idea that disrupted CRH-dependent intracellular signaling may contribute to the dynamic changes in CRH-evoked excitability observed. No significant change in expression of the AVP receptor (*Avpr1b*) or glucocorticoid receptor (*Nr3c1*) that are key determinants of controlling corticotroph physiology were observed although the GR chaperone *Fkbp5*, but not *Hsp90*, was significantly downregulated in CS-4R. However, this cluster also includes enrichment for components of several other signaling pathways important for corticotroph physiology, including members of the MAPK and Wnt signaling pathways (30, 31).

In contrast to this large subset of genes that were downregulated at CS-4R, other genes showed different dynamics of expression following withdrawal of the stress. A subset of genes (322 genes) was downregulated in both CS and/or CS-4R and expression was further suppressed at CS-12R. GO analysis of this cluster revealed no significant gene set enrichment (Fig. 5A-5C). A smaller number of genes (200) were significantly upregulated only 12 weeks following removal of the chronic stress (C12-R group), with significant enrichment for genes regulating lipid mobilization and transport. A very small cluster of 44 genes was significantly upregulated during CS or CS-4R but expression was largely returned to CTRL levels by CS-12R (Fig. 5A-5C). GO analysis of this cluster revealed enrichment for genes involved in cellular respiration including multiple components of the mitochondrial respiratory chain complex including subunits of ATP synthase (*mt*-*Atp*6), cytochrome c oxidase (*mt-Co*1, *mt-Co*3), cytochrome b (*mt-Cytb*), and NADH dehydrogenase (*mt-Nd1*).

**Figure 6.**
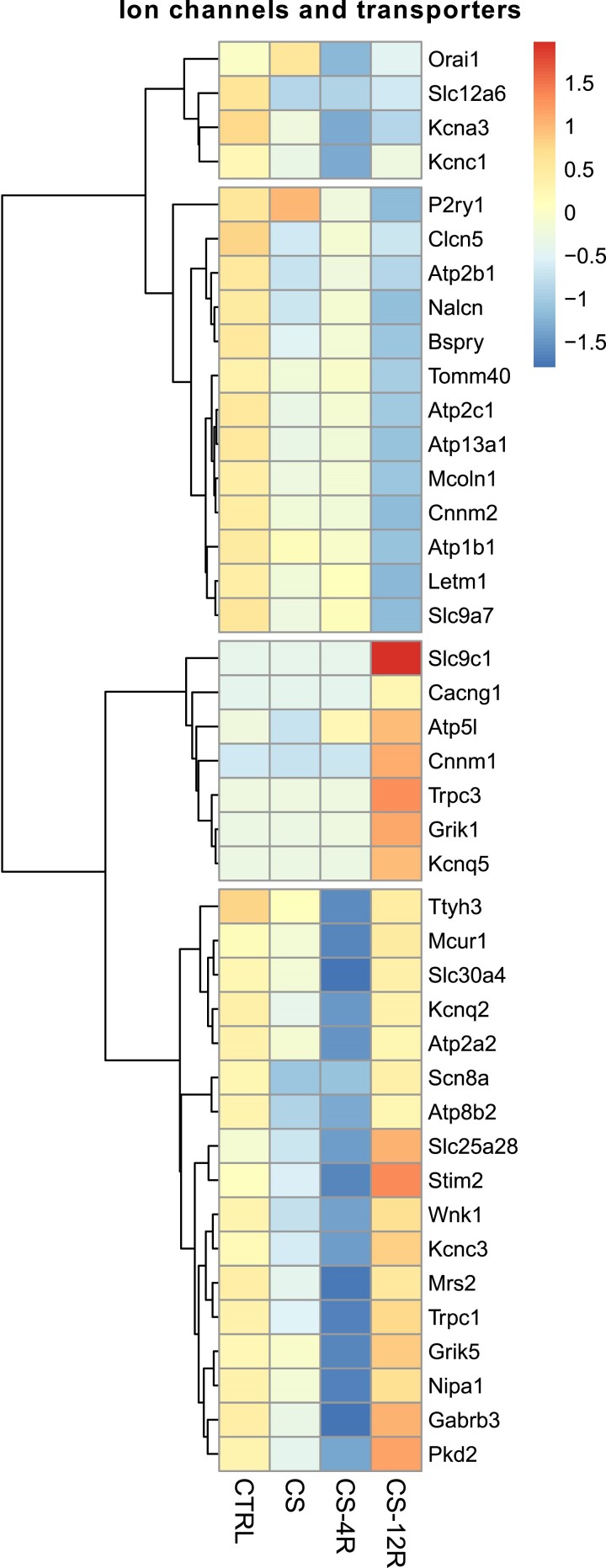
Ion channels and transporter transcription is modulated in stress and recovery. Corticotrophs from control (CTRL, n = 6 preps), chronic stress (CS, n = 3 preps), and stress plus 4- (CS-4R, n = 3 preps) or 12-week (CS-12R, n = 3 preps) recovery were FACS-sorted and the transcriptome analyzed by bulk RNA-seq. (A) Heatmap showing differentially expressed ion channels and transporters with relative expression across each row (*z*-score) is shown as per the color code.

Although genes encoding ion channels and transporters were not enriched in these clusters, 41 DEGs were ion channels/transporters representing 7.7% of the ion channels/transporters in the GO categories (Fig. 6). No significant change in mRNA expression of Kcnma1, the single gene that encodes the pore-forming subunit of BK channels, nor any of its known regulatory subunits expressed at low levels in our RNA-seq datasets (β-subunits encoded by: Kcnmb2, 3, and 4; γ-subunits encoded by Lrrc26, 38, 52, 55, or the Lingo1-4 family) was observed at CS-4R. This supports our electrophysiological data that a loss of BK channel expression per se likely does not underly the major suppression of corticotroph excitability at CS-4R, although a reduction in functional coupling to BK may contribute to this period of hypoexcitability. In this regard, multiple ion channels that are predicted to control spiking and bursting, including a range of voltage-gated potassium channels are differentially expressed at CS-4R compared to control (Fig. 6).

In addition, comparisons between CTRL and aged-CTRL cells revealed a very modest effect of age with 22 DEGs (6 downregulated, 16 upregulated DEGs with a log_2_ FC > 1) in aged-CTRL compared to CTRL (see Supplementary Fig. S1 (28)). Taken together, major dynamic changes in the transcriptional landscape of corticotrophs are associated with the dynamic changes in corticotroph electrical excitability that occur for weeks after cessation of a period of chronic stress. Multiple distinct mechanisms, with different dynamics and timescales likely control corticotroph excitability for weeks following a period of chronic stress.

## Discussion

Understanding the dynamics and mechanisms by which chronic stress remodels corticotroph physiology is critical to our ability to both diagnose and treat disorders of HPA dysfunction. Here, we reveal that rather than major changes in the transcriptional landscape of corticotrophs occurring in direct response to a period of chronic stress, these occur after the period of chronic stress has been removed and persist for weeks to months. It is often assumed that a key mechanism for driving changes in the physiology of key components of the HPA axis results from large-scale changes in gene expression in response to a period of chronic stress per se. However, this assumption is largely based on analysis of gene expression using brain tissue containing multiple cell types. Indeed, single cell RNA-seq analysis of the anterior pituitary from male mice exposed to 3 weeks of a chronic social defeat stress paradigm revealed 32 DEGs at the level of corticotrophs (similar to the 35 DEGs observed in our analysis of FACS purified corticotrophs) compared to >900 DEGs when summing across all pituitary cell types in the anterior pituitary. A similar order of magnitude number of DEGs has also been reported in hypothalamic tissue extracted from male mice subject to a range of different chronic stress paradigms. For example, chronic restraint stress revealed 108 DEGs (32); chronic variable stress revealed 114 DEGs (33); and chronic social defeat stress revealed 66 DEGs (16), although another study (34) using chronic social defeat show an order of magnitude higher number of DEGs.

Importantly, distinct patterns of differentially expressed genes are observed with the major response being a downregulation of genes 4 weeks after the period of chronic stress that largely recovers by 12 weeks. This major downregulation in gene expression 4 weeks after the period of repeated daily restraint chronic stress is associated with enrichment for genes involved at multiple levels of the cAMP/PKA as well as other signaling pathways. In contrast, other gene expression pathways are altered after longer recovery periods are revealing long-lasting changes in the corticotroph transcriptional landscape for months after an episode of chronic stress. This dynamic pattern at the transcriptional level is also paralleled with suppression of both basal and CRH-induced excitability 1 to 4 weeks after the period of chronic stress that only partial recovers over the following 4 months.

This reversible suppression of CRH-induced excitability, associated with downregulation of multiple components of the cAMP/PKA pathways, is likely a key contributing factor to the hypoexcitability of corticotrophs following a period of chronic stress. However, the extent to which this hypoexcitability contributes to a suppression of corticotroph function remains to be explored as multiple signaling, trafficking, and metabolic pathways are also modified in this period, as revealed by our RNA-seq analysis. Functionally, in humans, a major diagnostic for stress related disorders is the use of a CRH-stimulation test that reveals hyporesponsiveness of the anterior pituitary corticotrophs for several weeks after a period of chronic stress. Recent mathematical modelling has defined a decrease in corticotroph functional mass as a key determinant of this hyporesponsiveness (11). Our data suggest that this change in functional mass is not due to a reduction in corticotroph number, or size, as proposed (11). Rather, it results from a suppression of CRH-evoked electrical excitability that would be predicted to result in a reduced efficiency of CRH-induced stimulus-secretion coupling, an important determinant of corticotroph functional mass. Importantly, recent mathematical modelling also suggests a simple hysteresis state following a period of chronic stress leading to recovery of HPA axis function (35). Our data reveal complex changes in both excitability and corticotroph transcriptome landscape that suggest a more dynamic interplay rather than a simple toggle switch or hysteresis loop for “recovery” of corticotrophs following chronic stress. For example, we see a large number of genes whose expression changes only at 12 weeks following the period of chronic stress even though the majority of genes do follow the time course of changes in excitability that we see. In this regard, we do not know what the key drivers of these changes are in corticotroph gene expression. For example, it may arise from modified feed-forward drive from changes in CRH and/or AVP output from hypothalamic neurons, driven by changes in circulating glucocorticoid levels from the adrenal or a consequence of changes in intra-pituitary cellular interactions and networks. Interestingly, in multiple brain regions from male mice subjected to either chronic social defeat stress (36) or chronic repeated daily restraint stress (37), significant gene expression changes are still seen 3 to 4 weeks after cessation of the chronic stress with the expression profile largely distinct from that seen immediately after the period of chronic stress. This might support that some of the changes in dynamics we see at the corticotroph also result from different dynamics of other levels of the integrated HPA system.

In addition, we also observe differences in the proportions of cells that display CRH-induced bursting following chronic repeated restraint stress, in addition to reduced bursting in each cell. This suggests that chronic repeated restraint stress is also modifying the proportion of corticotrophs that display CRH-dependent bursting behavior. Intriguingly female mouse corticotrophs display a much smaller proportion of cells that display CRH-dependent bursting compared to males (21). Clearly, which other aspects of corticotroph function, beyond electrical excitability, are also modified or contribute to the dynamic changes we see, including hyporesponsiveness to the other major hypothalamic secretagogue, AVP; enhanced glucocorticoid feedback; changes in mitochondrial metabolism or reduced ACTH secretory capacity remains to be determined. In this context, although AVP1b receptor mRNA is not changed in our model at CS-4R, both Fkbp5 and Creb3l1 are downregulated. Genetic deletion of the glucocorticoid receptor chaperone Fkbp5 enhances glucocorticoid feedback at the level of the mouse pituitary (38) and we observe no change in mRNA expression of hsp90 or GR itself. Creb3l1 is important for maintaining secretory capacity in many neuroendocrine cells (39, 40), suggesting that other components of the secretory machinery may be affected. However, the lack of suppression of Creb3l2 expression, which also regulates secretory capacity and increases corticotroph cell size by acting as a translation scaling factor (41), further supports that changes in cell size do not contribute to the changes in excitability we observe.

Importantly, a goal for the future is to establish whether the dynamics and extent of changes in both electrical excitability and gene expression that we observed for weeks following a 14-day exposure to repeated daily restraint stress are also manifest in other chronic stress paradigms. In this study, we used a homotypic model of repeated daily restraint stress in mice that typically displays a modest, but incomplete, habituation of the HPA axis to the repeated homotypic stressor as determined by analysis of ACTH and/or CORT output (17). Habituation of the HPA axis is a common, but not universal, feature of chronic stress paradigms using a typical homotypic processive stressor in mice, rats, and humans (42-46) and is therefore likely a normal physiological response as chronic stress progresses. In rodents and humans, habituation to repeated processive stressors has been shown to involve changes in higher brain centers (44, 47-49). However, habituation is generally less pronounced with more physical stressors, and considerable variations in habituation may result from the duration and frequency of repeated stress exposure as well as the species, strain, and context of stress paradigms under investigation (47-49). Habituation has been reported to persist following repeated restraint stress in rats for several weeks after the stressor has been removed (47). In formal definitions of habituation in neurones, a response lower than baseline and facilitation prior to a suppression of the response may be evident although formal testing of this in the HPA is largely lacking (42, 43, 47). As such, we cannot exclude that the dynamic changes we observe in both corticotroph excitability and gene expression after repeated restraint stress in mice result from potential mechanisms of HPA axis habituation to a homotypic stressor rather than other physiological mechanisms such as desensitization (50). For example, immediately following the 14 days of daily restraint in mice, we see an enhanced, rather than suppressed, spontaneous and CRH-induced excitability of corticotrophs with only a small change in corticotroph gene expression. In support of this facilitation, previous work using a 14-day chronic variable stress paradigm in male rats (13) revealed enhancement of basal and CRH-induced ACTH release in isolated pituitaries in vitro in the chronic stress group compared to control. This suggests potential common mechanisms between species and both heterotypic and homotypic chronic stress paradigms at the level of the corticotroph in rodents. Second, both the spontaneous and CRH-evoked excitability of corticotrophs at 4 weeks after recovery from the chronic stress paradigm shows a suppression below that observed in control corticotrophs, before the period of chronic stress, in parallel with large-scale changes in corticotroph gene expression that may be linked either to long-term habituation or desensitization. In general, habituation would not typically be predicted to reduce basal activity only the stimulated activity a property more aligned to desensitization (50). Regardless of whether the changes in excitability and gene expression observed results from mechanisms of habituation at the level of the corticotroph per se, the dramatic changes in both excitability and gene expression observed 4 weeks of recovery after the period of chronic stress reveals that suppression of corticotroph function is a major contributor to the long-term suppression of HPA axis activity during recovery from a period of chronic stress. Clearly, an important question for the future is the extent to which the duration and nature (eg, homotypic vs heterotypic) of the stressor used during the chronic stress period as well whether habituation, sex, species, or context are key determinants of the dynamics and mechanisms during recovery in corticotroph regulation and physiology for potential translational of these findings.

In conclusion, this study provides new insight into the dynamics of HPA axis function in defining mechanisms that modify corticotroph physiology following a period of chronic stress. Importantly, long-term dynamic changes in both corticotroph transcriptome and electrical excitability are manifest for weeks in the period following cessation of a period of chronic restraint stress. These insights provide a new framework to further understand underlying molecular mechanisms as well approaches to both diagnosis and treatment of stress-related dysfunction of the HPA axis.

## Data Availability

Original data generated and analyzed during this study are included in this published article or in the data repositories listed in References.

## Abbreviations

BK: large conductance calcium- and voltage- activated potassium channel
CRH: corticotrophin-releasing hormone
CS: chronic stress
CTRL: control
DEG: differentially expressed gene
FACS: fluorescence-activated cell sorting
GFP: green fluorescent protein
GO: gene ontology
HPA: hypothalamic-pituitary-adrenal
PKA: protein kinase A
POMC: proopiomelanocortin
RNA-seq: RNA sequencing
